# Roughage quality determines the production performance of post-weaned Hu sheep via altering ruminal fermentation, morphology, microbiota, and the global methylome landscape of the rumen wall

**DOI:** 10.3389/frmbi.2023.1272625

**Published:** 2024-01-03

**Authors:** Sen Ma, Yan Zhang, Zidan Li, Ming Guo, Boshuai Liu, Zhichang Wang, Yalei Cui, Chengzhang Wang, Defeng Li, Yinghua Shi

**Affiliations:** ^1^ Department of Animal Nutrition and Feed Science, College of Animal Science and Technology, Henan Agricultural University, Zhengzhou, China; ^2^ Henan Key Laboratory of Grassland Resources Innovation and Utilization, Henan Agricultural University, Zhengzhou, China; ^3^ Henan Herbage Engineering Technology Research Center, Henan Agricultural University, Zhengzhou, China

**Keywords:** Hu sheep, roughage quality, rumen fermentation, papillae development, rumen microbiome, rumen wall methylome

## Abstract

Roughage quality is a crucial factor influencing the growth performance and feeding cost of ruminants; however, a systematic investigation of the mechanisms underlying this is still lacking. In this study, we examined the growth performance, meat quality, ruminal fermentation parameters, rumen microbiome, and tissue methylomes of post-weaned Hu sheep fed low- or high-quality forage-based diets. Our results showed that sheep in the alfalfa hay (AG) and peanut vine (PG) groups exhibited better growth performance, slaughter performance, and meat quality than sheep in the wheat straw group (WG). The sheep in the AG possessed relatively higher contents of serum immunoglobins (IgA, IgG, and IgM) and lower contents of serum inflammation factors (TNF-α, IL-1β, IL-6, and IL-8) than those in the WG and the PG did. In addition, the levels of blood T lymphocytes (CD4^+^ and CD8^+^) and the CD4-to-CD8 ratio were significantly higher in the AG sheep than in the WG sheep and PG sheep. The concentration of ruminal NH_3_-N was highest in WG sheep, whereas the concentrations of individual and total short-chain fatty acids (SCFAs) were highest in the PG sheep. The length, width, and surface area of ruminal papillae were markedly different among the three groups, with the sheep in the PG being the most morphologically developed. The main ruminal microbes at the genus level include *Prevotella 1*, *Rikenellaceae RC9 gut group, norank f F082*, *Ruminococcus 1*, and *Ruminococcus 2*. The relative abundances of certain species are positively or negatively associated with fermentation parameters and growth index. For example, the fibrolytic bacteria *Ruminococcaceae UGG-001* showed positive relationships with the concentration of SCFAs, except propionate. In addition, the relative abundances of fibrolytic bacteria (e.g., *Ruminoccus 1*) showed a negative relationship with starch-degrading bacteria (e.g., *Prevotellaceae*). The genome-wide DNA methylation analysis revealed that rumen tissues in the PG sheep and WG sheep occupied different global DNA methylomes. The genes with differentially methylated promoters were involved in known pathways (e.g., the FoxO signaling pathway) and the Gene Ontology (GO) terms (e.g., anatomical structure morphogenesis) pertaining to rumen development. Two candidate genes (*ACADL* and *ENSOARG00020014533*) with hyper- and hypo-methylated promoters were screened as potential regulators of rumen development. In conclusion, roughage quality determines sheep growth performance via directly influencing rumen fermentation and microbiome composition, and indirectly affecting rumen development at the epigenetic level.

## 1 Introduction

Ruminants, including sheep and goats, are a unique class of herbivores with an outstanding ability to transform human-inedible fibrous feedstuffs and industrial byproducts into high-quality animal-sourced products (e.g., meat and milk) (Pulina et al., 2017). Typically, ruminant diets are composed mainly of concentrates, silage, roughage, and other feed additives. In modern intensive production systems, roughage, which is mostly fibrous plant-derived feed ingredients, usually constitutes approximately 20%–60% of the dry matter weight in a conventional ruminant ration (Suárez et al., 2007; Malik et al., 2020; Yi et al., 2022). Thus, the characteristics and prices of roughage are not only the key determinants influencing the production performance and product quality of ruminants, but also the pivotal factors affecting livestock rearing profit. For example, Zhu et al. (2022) fed fattening steers with wheat straw-, alfalfa hay-, and peanut vine-based diets, and discovered that the cattle eating high-quality fodders (i.e., alfalfa hay and peanut vine) exhibited better production performance and meat quality than those consuming low-quality forage (i.e., wheat straw). Similar results have been extensively reported in other ruminants with diverse production purposes, including dairy cows (Wang et al., 2014), sheep (Obeidat et al., 2019), and goats (Hwang et al., 2018). However, the adequate supply and lower price of crop straws represent their unparalleled advantages over high-quality fodders in ruminant production. Mechanistic explorations of the differences between ruminants fed fodders with distinct qualities or features are crucial for the sufficient utilization of forage resources and highly efficient ruminant production (Beigh et al., 2017).

The sufficient utilization of fibrous forages by ruminants relies on the rumen, the largest anaerobic stomach chamber, which harbors a complex and dynamic functional microbe community that plays decisive roles in the efficient biodegradation of ingested feed and synthesis of microbially derived nutrients (Membrive, 2016). Previous studies have explored the ruminal microbial differences between animals fed low-quality and high-quality forage-based diets, and found dramatic variations in the abundances of core rumen microbes with characteristics that are important for the decomposition of structural and non-structural carbohydrates (Wei et al., 2021; Zhu et al., 2022; Eberly et al., 2023). For example, Liu et al. found higher proportions of the fiber-degrading *Butyrivibrio* and *Prevotella* in the alfalfa diet than in the rice straw diet, and a reverse pattern of unclassified *Ruminococcaceae* (Liu et al., 2016). However, the majority of these studies have focused on the changes of individual microbial species in ruminants fed diverse forage types. Few studies have unveiled the interaction relationships of different microbial groups inside the rumen, even though there is much evidence pointing out that widespread cooperative or competitive relationships exist among ruminal microbes (Solden et al., 2018; Won et al., 2020). At the same time, a deeper understanding of the interactions among ruminal microbes has been proposed as a helpful method for optimizing the rumen fermentation ecosystem and developing novel strategies for the efficient utilization of poor-quality fodders (Wadhwa et al., 2016).

In addition to colonized microbes, the adequate tissue development of rumen itself is a vital factor affecting production efficiency. The absorption of fermented final products [i.e., short-chain fatty acids (SCFAs)], vitamins, minerals, and the other nutrients necessary for host utilization is determined by the rumen wall and its associated tissue protrusions, the rumen papillae (Baldwin and Connor, 2017). The existence of rumen papillae endow the rumen with a larger surface area and stronger ability for nutrient assimilation; thus, the height and width of the rumen papillae are important indicators of rumen development. Previous studies have suggested that rumen papillae development is exclusively driven by SCFAs (especially butyrate) and is further controlled by diet composition (Gupta et al., 2016; Baldwin and Connor, 2017; Niwińska et al., 2017). However, the molecular mechanisms underlying the stimulatory effects of SCFAs on rumen papillae development are not yet well understood. The DNA methylation-mediated epigenetic modulation of gene expression by SCFAs has been widely reported in humans and mice (Lu et al., 2018; Guo et al., 2022). In addition, a recent study suggested that global rumen tissue methylation profiles significantly differed between grass-fed and grain-fed Angus cattle (Li et al., 2019). These studies indicate that SCFA-regulated DNA methylation could be a pivotal aspect influencing rumen development.

Although individual studies have proven that the quality of fodder markedly determines animal production performance via affecting the rumen microbiome, fermentation, and morphology, a systematic exploration of microbial and host responses is still lacking, especially of rumen microbial interactions and the interplay between SCFAs and rumen epigenomes. In this study, we assessed the growth performance, health index, and rumen microbiomes of post-weaned Hu sheep fed high- or low-quality forage-based diets. We also examined the rumen tissue morphology and methylome to dissect the epigenetic mechanisms related to rumen development. Our study revealed the complex microbial and host acclimatation to diets with differential fodder quality, which is helpful for intentionally developing new feed rations, probiotics, additives, and other methods to improve forage utilization and ruminant production efficiency.

## 2 Materials and methods

### 2.1 Farm animals, experimental design, and rearing management

Sixty 3-month-old post-weaned male Hu sheep with a healthy body condition, normal appetite, and similar body weight (24 kg–28 kg) were selected as the experimental animals. The animals were then randomly divided into three groups: the wheat straw group (WG), the alfalfa hay group (AG), and the peanut vine group (PG). Each group contained four replicates, and five sheep were included in one replicate. The nutritive information of the forage used in this study is provided in **Table 1**. The feed composition and nutritive values of diet in each group are listed in **Table 2**. The experiment was subdivided into a 7-day pre-trial stage and a 60-day trial stage. The diet nutritive levels were set in accordance with the Chinese industrial feeding standard of meat-producing sheep (NY/T 816-2004). All feedstuffs were mixed uniformly in a total mixed ration (TMR) machine and utilized instantly after mixing.

**Table 1:** Nutritive value of three types of forage.

Item	Wheat straw	Alfalfa hay	Peanut vine
DM, %	89.6	91.3	92.4
DE, MJ/kg	4.91	7.83	7.24
CP, %	5.60	15.1	8.57
EE, %	1.80	2.50	1.30
NDF, %	81.00	39.69	45.00
ADF, %	58.00	31.99	35.00
Ca, %	0.17	1.24	1.25
P, %	0.05	0.26	0.40

DM, dry matter; DE, digestive energy; CP, crude protein; EE, ether extract; NDF, neutral detergent fiber; ADF, acid detergent fiber.

**Table 2:** Composition and nutritive value of the diets with distinct forages (on a DM basis).

Item	WG^1^	AG^1^	PG^1^
Ingredients (%)			
Corn (4-07-0280)	38.4	37.4	36.4
Soybean meal	18	10	12
Bran (4-08-0069)	6	6	6
Distiller’s grains	2	11	9
Wheat straw	30	0	0
Alfalfa hay	0	30	0
Peanut vine	0	0	30
Limestone	1.5	1.5	1.5
Salt	0.5	0.5	0.5
Sodium phosphate	0.6	0.6	0.6
Premix^2^	3	3	3
Total	100	100	100
Nutritional levels^3^ (DM)			
DE/(MJ/kg)	10.30	10.35	10.22
CP, %	16.36	16.36	16.35
NDF, %	35.03	25.84	26.53
ADF, %	22.53	17.93	18.13
Ash, %	4.69	4.97	5.25
Ca, %	1.54	1.65	1.65
P, %	0.48	0.51	0.56

^1^WG, wheat straw group; AG, alfalfa hay group; PG, peanut vine group.

^2^Per kg, the premix contained: vitamin A, 300,000 IU; vitamin D_3,_ 100,000 IU; vitamin E, 300 IU; Fe, 1,500 mg; Cu, 750 mg; Zn, 400 mg; Mn, 4,500 mg; I, 240 mg; Se, 16 mg; Co, 55 mg; Ca, 223 g; and P, 40 g.

^3^DE was a calculated value, which was calculated according to the tables of feed composition and nutritive values in China (version: 31). The other nutrient levels are measured levels.

The daily management of the experimental animals was performed as usual, except that the conventional feed was gradually substituted by the diet we mentioned above in the pre-trial stage. During the 60-day trial stage, the experimental animals were fed twice a day (at 08:30 and 16:30) in an *ad libitum* manner, and they were also given free access to water. The animals’ daily feed intake (DFI) was recorded over the experimental period, and the ruminal fluid from one animal of each replicate was collected at the end of the feeding experiment, as was previously described (Zhu et al., 2022). In addition, the livestock status, including the animals’ appetite, water intake, and feces morphology, was monitored to ensure they were in healthy condition.

### 2.2 Measurement of production performance

The average daily feed intake [ADFI, on a dry matter (DM) basis] was calculated by determining the difference between the weight of the feed provided to the animals and the weight of the residual feed for 1 day. The live weights of sheep were measured in the morning before providing feed and water at the first day and 60th day of the experiment. The total weight gain (kg) was calculated as the difference between the initial body weight (as measured on the first day) and the final weight (as measured on the 60th day). The average daily gain (ADG, g/d) was calculated by dividing the total weight gain (g) value by the duration of the experiment (60 days). The feed-to-gain (F/G) ratio was calculated by dividing the ADFI value by the ADG value.

### 2.3 Measurement of immunological parameters

On the 60th day of this experiment, four sheep from each group were selected, and 10 mL of blood was collected using a vacutainer from venous vessels in the neck. Subsequently, blood serum was obtained using a conventional method, that is, clotting at room temperature for 15 min, and centrifuging at 3,000 rpm for 15 min. After that, the serum was transferred into a 1.5-mL centrifuge tube and stored at a temperature of −20°C for later use. The serum levels of immunoglobin A (IgA), immunoglobin G (IgG), and immunoglobin M (IgM) were determined using a fully automatic biochemical analyzer (AU680, Beckman, CA, USA). The abundances of interleukin 1 beta (IL-1β; H002-1-2), interleukin 6 (IL-6; H007-1-1), interleukin 8 (IL-8; H008-1-1), tumor necrosis factor alpha (TNF-α; H052-1-2), interferon gamma (IFN-γ; H025-1-2), CD4 (H156-1-1), and CD8 (H157-1-1) were analyzed using the enzyme-linked immunosorbent assay (ELISA) method. All assay kits were purchased from Nanjing Jiancheng Bioengineering Institute (Nanjing, China). The experimental procedure was performed in accordance with the manufacturer’s guidelines.

### 2.4 Measurement of slaughter performance

On the 60th day of this study, one animal from each replicate was selected so that the slaughter performance of the sheep fed with the abovementioned three diet types could be assessed. The procedures of sample collection, slaughter performance examination, and meat quality determination (longissimus dorsi) were performed as previously reported (Jia et al., 2022).

### 2.5 Determination of rumen fermentation parameter and morphology

After sacrifice, the compound stomach (including the rumen, reticulum, omasum, and abomasum) of the sheep was removed and washed to eliminate the attached chyme. Tissue samples measuring 2 cm × 2 cm in the middle of the ventral sac of the sheep rumen were harvested and stored in 10% neutral formalin solution for fixation. The measurement of the length, width, and surface area of ruminal papillae, and the thickness of submucosa, muscular, and mesothelium layers was carried out as reported before (Álvarez-Rodríguez et al., 2012).

The measurement of ruminal pH was performed instantly using a portable pH meter (Testo 206-pH2; Testo AG, Baden-Wurttemberg, Germany) after the sheep were sacrificed. Meanwhile, the ruminal fluid was filtered using four-layered gauze and stored at −20°C. The ruminal concentration of NH_3_-N was measured using the classic phenol–sodium hypochlorite colorimetric method (Broderick and Kang, 1980). The concentration of SCFAs in the ruminal fluid was assessed using a high-performance liquid chromatography (HPLC)-based method (Isac et al., 1994).

### 2.6 Ruminal bacteria DNA extraction, PCR amplification, and Illumina MiSeq sequencing

The total DNA was extracted from rumen fluid samples using an E.Z.N.A.^®^ Stool DNA Kit (Omega Bio-Tek, Norcross, GA, USA). The V3 to V4 hypervariable region of the 16S rRNA gene was amplified using PCR with the following thermocycling protocol: 95°C for 2 min, followed by 30 cycles at 95°C for 20 s, 55°C for 30 s, and 72°C for 30 s, followed by a final extension stage at 72°C for 5 min. The primers used to amplify the sequences were as follows: V338F (5′-ACTCCTACGGGAGGCAGCAG-3′ and V806R (5′-GGACTACHVGGGTWTCTAAT-3′). The PCR reactions were performed in triplicate for 20-μL reactions consisting of 2 μL of 10 × FastPfu Buffer, 2 μL of 2.5 mM dNTPs, 0.8 μL of each primer (5 mM), 0.2 μL of FastPfu polymerase (TransGen Biotech, Beijing, China), 200 nM of each primer (Majorbio, Shanghai, China), and 10 ng of template DNA. The gel fragments of the correct size were excised and purified using an AxyPrep™ DNA gel extraction kit (Axygen, Union City, NJ, USA). The purified amplicons were equimolar pooled and paired-end sequenced (2 bp × 300 bp) on an Illumina MiSeq platform in accordance with standard protocols. All experimental procedures were performed in accordance with the manufacturer’s instructions. The raw reads were deposited into the National Center for Biotechnology Information (NCBI)’s Sequence Read Archive (SRA) database (accession number: PRJNA991257).

### 2.7 Sequencing data processing

The raw 16S rRNA gene sequencing reads were demultiplexed, quality filtered using fastp (version 0.20.0) (Chen et al., 2018), and merged using FLASH (version 1.2.7) (Lozupone and Knight, 2005) in accordance with the following criteria: (i) the 300-bp reads were truncated at any site receiving an average quality score of < 20 over a 50-bp sliding window, the truncated reads shorter than 50 bp were discarded, and the reads containing ambiguous characters were also discarded; (ii) only overlapping sequences longer than 10 bp were assembled according to their overlapped sequence; the maximum mismatch ratio of the overlap region was 0.2, and the reads that could not be assembled were discarded; and (iii) the samples were distinguished according to the barcode and primers, and the sequence direction was adjusted. The operational taxonomic units (OTUs) meeting the 97% similarity cutoff value were clustered using UPARSE version 7.1 (Edgar, 2013), and the chimeric sequences were identified and removed. The taxonomy of each representative sequence was analyzed using the RDP Classifier (version 2.11, http://sourceforge.net/projects/rdp-classifier/) against the 16S rRNA database (SILVA, version 138) using a confidence threshold of 0.7.

### 2.8 Correlation analysis among ruminal fermentation parameters, growth parameters, and the microbiome data

Pearson’s correlation analysis was performed using the online Majorbio platform (https://www.majorbio.com). The results were visualized using the R pheatmap package (version: 1.0.12). A *p*-value < 0.05 was set as significant for all analyses.

### 2.9 Phylogenetic tree construction, visualization, and microbial functional prediction

FastTree (version 2.1.3, http://www.microbesonline.org/fasttree/) was selected to construct the phylogenetic tree of the microbes at the genus level based on OTU data. The neighbor-joining method was adopted in the present study, and the result was visualized using R (version 3.3.1). The functional prediction of microbes was performed using the online tool PICRUSt2 (Douglas et al., 2020) on the Majorbio platform.

### 2.10 Methylome analysis of the ruminal epithelial tissues and bioinformatic analysis

Reduced representation bisulfite sequencing (RRBS) was used to profile the methylome of the ruminal tissues in accordance with the standard protocol developed by Novogene Co., Ltd (Beijing, China). In brief, ruminal epithelial tissues were collected and rinsed twice to the remove the bacteria and adherent feedstuffs. Subsequently, the genomic DNA was extracted using the mammalian genomic DNA extraction kit (D0061, Beyotime, Beijing, China). A total of 1.5 μg of genomic DNA spiked with moderate lambda DNA was digested by *Msp*I, after which it was subjected to end repair and adenylation. The cytosine-methylated barcodes were ligated to the DNA as per the manufacturer’s instructions. These DNA fragments were then treated with bisulfite using the EZ DNA Methylation-Gold™ Kit (Zymo Research). The sequencing libraries were constructed by Novogene Co., Ltd (Beijing, China). Subsequently, the paired-end sequencing of the samples was performed using the Illumina platform and 150-bp paired-end reads were generated (Illumina, CA, USA).

Subsequently, the obtained raw reads were assessed using FastQC (fastqc v0.11.5) and fastp (fastp 0.20.0) to acquire the filtered clean reads. The reads were then mapped to the sheep reference genome (ARS-UI Ramb v1.0) using Bismark software (version 0.16.3) (Krueger and Andrews, 2011). The reference genome was first transformed into a bisulfite-converted version (C-to-T and G-to-A converted) and then indexed using bowtie2 (Langmead and Salzberg, 2012). The clean reads were also transformed into fully bisulfite-converted versions (C-to-T and G-to-A converted) before being aligned to the similarly converted versions of the genome in a directional manner. The sequence reads that produced a unique best alignment from the two alignment processes (original top and bottom strand) were then compared with the normal genomic sequence, and the methylation state of all cytosine positions was inferred. The same reads that aligned to the same regions of genome were regarded as duplicates. The sequencing depth and coverage were summarized using the deduplicated reads. The results of the methylation extractor (Bismarck methylation extractor—no overlap) were transformed into the bigWig format for visualization. The sodium bisulfite non-conversion rate was calculated as the percentage of cytosines sequenced at the cytosine reference positions in the lambda genome. The methylated sites were identified by way of a binomial test using the methylated counts (mC), total counts (mC + umC), and the non-conversion rate (r). The sites with an FDR-corrected *p*-value < 0.05 were considered methylated sites. To calculate the methylation level of the sequence, we divided the sequence into multiple bins, with a bin size 10 kb. The sum of methylated and unmethylated read counts in each window was calculated. The methylation level (ML) for each window or C site shows the fraction of methylated Cs, and is defined as follows:


(1) ML(C)=reads(mC)/reads(mC)+reads(C).

Differentially methylated regions (DMRs) were identified using the DSS software (Park and Wu, 2016). According to the distribution of DMRs throughout the genome, we defined the genes related to DMRs as the genes whose gene body region (from TSS to TES) or promoter region (2 kb upstream from the TSS) overlapped with the DMRs. The sequencing data were deposited in NCBI (accession number: PRJNA991642).

Gene Ontology (GO) enrichment analysis of genes related to DMRs was implemented using the GOseq R package (Young et al., 2010), in which the gene length bias was corrected. The GO terms with a *p*-value < 0.05 were considered significantly enriched by the differentially methylated promoter-related genes. We used KOBAS 3.0 software (Xie et al., 2011) to test the statistical enrichment of differentially methylated promoter-related genes in the Kyoto Encyclopedia of Genes and Genomes (KEGG) pathways.

### 2.11 Statistical analysis

The data in the present study are presented as mean ± standard deviation (SD). One-way ANOVA was adopted to determine the statistical significance among the groups, and Duncan’s multiple comparison was used to discern the differences in the means among the three groups. A two-tailed Student’s *t*-test was used to compare the differences in the means between the two groups. A *p*-value < 0.05 was considered statistically significant.

## 3 Results

### 3.1 Roughage quality markedly affects the growth performance and meat quality of post-weaned Hu sheep

**Figure 1:**
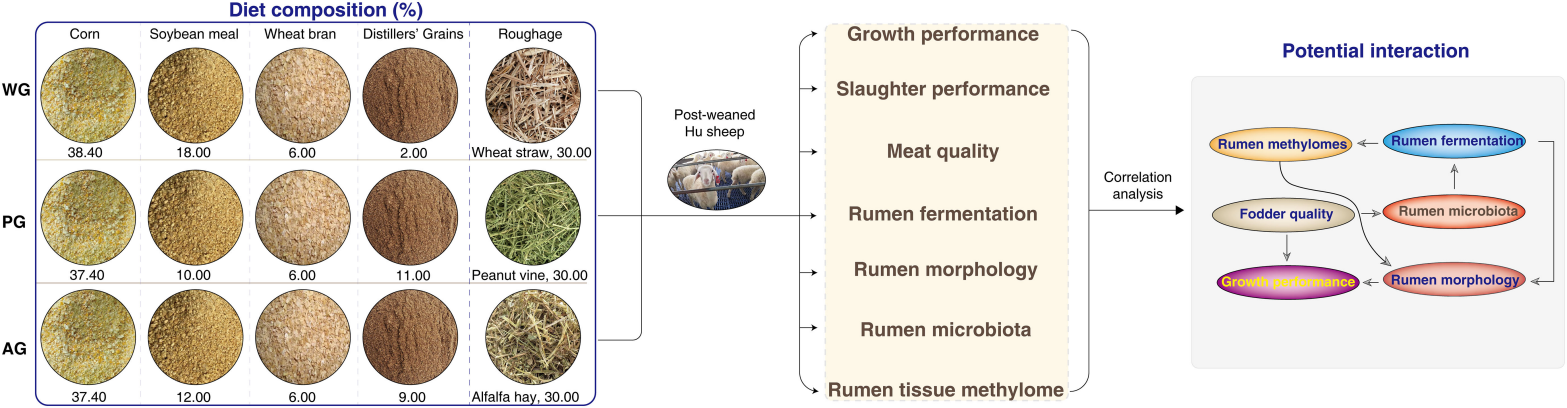
Schematic representation of the experimental design and data analysis.

**Figure 2:**
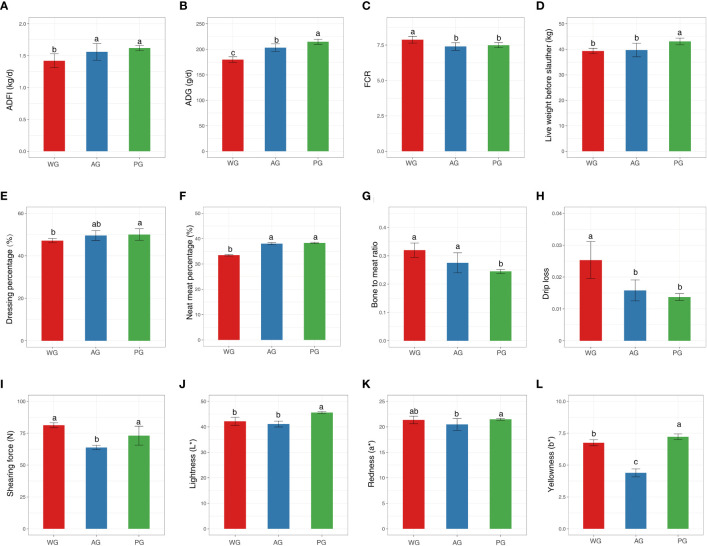
**(A–C)** Growth performance indices, **(D–G)** slaughter performance indices. **(H–L)** meat quality indices of post-weaned Hu sheep fed low- and high-quality roughage-based diets. ADFI, average daily feed intake; ADG, average daily gain; FCR, feed conversion ratio.

To examine the impacts of roughage quality on the production performance of post-weaned Hu sheep, we assessed the growth performance, slaughter performance, and meat quality of animals fed low-quality (wheat straw) or high-quality (alfalfa hay and peanut vine) roughage-based TMR diets with nearly identical crude protein and digestive energy (DE) levels (**Table 1**). The overall experimental design and associated downstream analysis are displayed in **Figure 1**. As shown in **Figure 2A**, the ADFI of sheep in the wheat straw group (WG) was significantly lower than those of the animals in the alfalfa hay group (AG) and the peanut vine group (PG). Consequently, the ADG of animals in the WG was the lowest among the three groups, with sheep in the PG possessing the highest ADG (**Figure 2B**). Meanwhile, sheep in the AG and the PG displayed markedly lower levels of FCR than animals in the WG (**Figure 2C**). The above results suggest that animals fed high-quality roughage exhibit superior growth compared with those fed low-quality forage.

Subsequently, we demonstrated that the live weight of animals before slaughter was heavier in the PG than in the WG and AG (**Figure 2D**), with there being no significant difference existing between the WG and the AG (*p* > 0.05). Similarly, the dressing and net meat percentages of animals in the PG displayed elevated levels compared with those of animals in the WG (**Figures 2E, F**). At the same time, among the three groups, the bone-to-meat ratio of slaughtered animals was lowest in the PG (**Figure 2G**). Moreover, no significant differences were found in the internal organ index (e.g., heart), GR values, and lion eye muscle areas of slaughtered animals among the groups (**Supplementary Tables 1 and 2**). These results indicated that the quality of the forage significantly affects the slaughter performance of post-weaned Hu sheep.

Finally, we tested the parameters pertaining to meat (longissimus dorsi) quality to dissect the impact of roughage grade on the eating characteristics of meat. As shown in **Figure 2H**, the drip loss of meat in sheep in the AG and PG was markedly lower than that of sheep in the WG (*p* < 0.05). The meat of sheep in the AG and PG was significantly more tender than that of sheep in the WG (measured by shearing force), although no statistical difference exists between the PG and WG (**Figure 2I**). In addition, among the three groups, the lightness (L*), redness (a*), and yellowness (b*) indices of the meat were highest in the PG, with slight differences observed between the WG and AG (**Figures 2J–L**). However, the pH_45min_, cooked meat yield, and water-holding capacity of the meat were similar among the groups (**Supplementary Table 3**). Collectively, these observations hinted that the roughage type exerts a substantial impact on the meat quality (i.e., eating quality and visual appearance) of Hu sheep.

### 3.2 Roughage quality influences the immune and inflammation status of post-weaned Hu sheep

**Figure 3:**
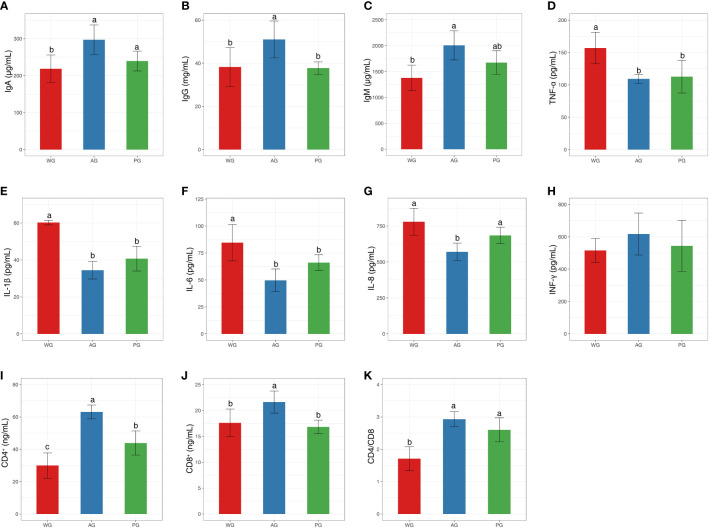
Immune and inflammation index values of post-weaned Hu sheep fed low- and high-quality roughage-based diets. **(A–C)** The levels of immunoglobulins in serum. **(D–H)** The levels of pro- and anti-inflammation factors in serum. **(I–K)** The levels of CD4^+^ and CD8^+^ leukocytes, and CD4/CD8 ratio in serum. Immune and inflammation index values of post-weaned Hu sheep fed low- and high-quality roughage-based diets. Ig, immunoglobulin; TNF-α,tumor necrosis factor-a; IL, interleukin; INF-γ, interferon gamma.

To clarify the role of roughage quality on the health status of post-weaned Hu sheep, we determined the abundances of immunoglobulins, inflammation factors, and immune cells from peripheral blood. The levels of IgA, IgG, and IgM were significantly higher in the AG than in the PG and WG, with no statistical differences existing between the PG and WG (**Figures 3A–C**). The protein abundances of pro-inflammatory factors (i.e., TNF-α, IL-1β, IL-6, and IL-8) were significantly lower in the PG and AG than in the WG, with their levels in the AG being the minimum (**Figures 3D–G**). At the same time, the level of INF-γ in the AG is higher than those of WG and PG, even did not reach statistical significance standard (*p* > 0.05, **Figure 3H**). Moreover, the numbers of CD4^+^ T lymphocytes ranked as follows: AG, PG, and WG (**Figure 3I**). However, the counts of CD8^+^ T lymphocytes were significantly higher in the AG than in the PG and WG (**Figure 3J**). The levels of CD4/CD8 were higher in the AG and PG than in the WG (**Figure 3K**). Notably, the quantities of CD4^+^ and CD8^+^ T lymphocytes, and CD4-to-CD8 ratio in the AG ranked first among the three groups. Taken together, these data clearly suggested that the fodder quality directly affects the partial key health status parameters of sheep, with the high-quality forage—alfalfa hay—exerting an optimally beneficial impact on the body condition of post-weaned sheep.

### 3.3 Roughage quality significantly impacts the ruminal fermentation parameters and tissue morphology of the sheep

**Figure 4:**
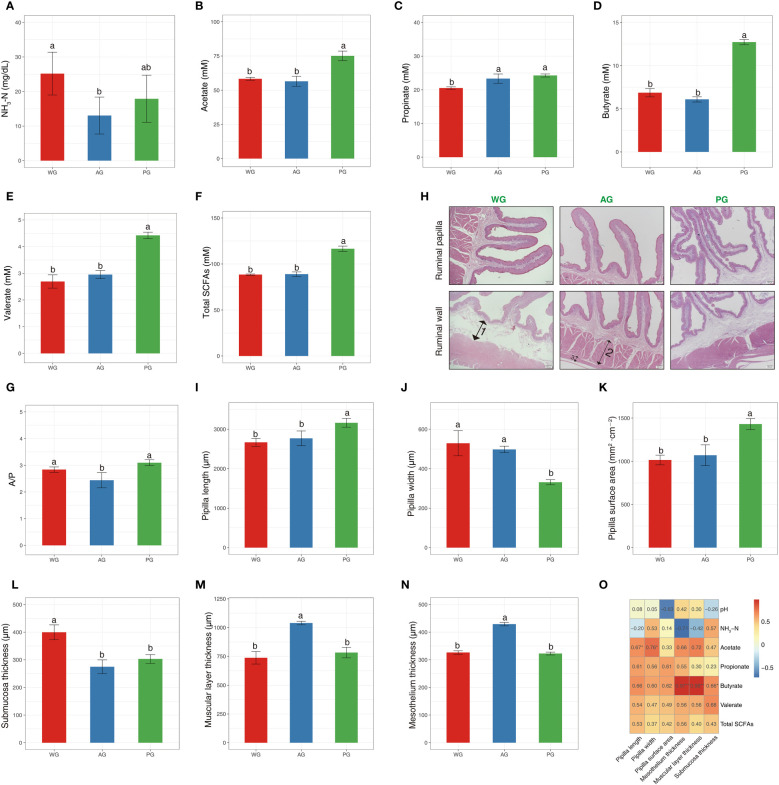
Rumen fermentation parameters and morphology of post-weaned Hu sheep fed low- and high-quality roughage-based diets, and Pearson’s correlation analysis. **(A–G)** Rumen fermentation parameters. **(H)** H&E staining of the ruminal wall. 1, submucosa; 2, muscular layer; 3, mesothelium. **(I–N)** Measurement of the index related to rumen morphology. **(O)** Pearson’s correlation analysis of rumen fermentation parameters with the index of rumen morphology. *, *p* < 0.05; **, *p* < 0.01. SCFAs, short-chain fatty acids.

To further determine the influence of roughage quality on rumen fermentation and animal growth, we analyzed several parameters from ruminal fluids and rumen tissues. The pH of ruminal fluids in the three groups were nearly identical (**Supplementary Table 4**). However, the concentration of NH_3_-N significantly differed among the three groups, with that in WG sheep being the highest and that in AG sheep being the lowest (**Figure 4A**). The concentrations of SCFAs (acetate, propionate, butyrate, valerate, and total SCFAs) were higher in the PG than in the WG and AG (**Figures 4B–F**), whereas the ratios of A/P were lowest in the AG, and highest in the PG (**Figure 4G**). The ruminal papillae in the PG were dramatically longer than they were in the WG and AG (**Figures 4H, I**). However, the papillae width possessed an adverse trend compared with the papillae length within the groups (**Figure 4J**). The surface area of ruminal papillae was larger in the PG than in the WG and AG (**Figure 4K**). In addition, the rumen wall morphology was markedly influenced by forage quality, in that the submucosa of WG sheep was thicker than that of AG sheep and PG sheep (**Figure 4L**). The thicknesses of the muscular layer and mesothelium were greater in the AG than in the PG and WG (**Figures 4M, N**). Pearson’s correlation analysis results indicated that the pH of ruminal fluid was negatively associated with the papillae surface area (**Figure 4O**). The concentration of NH_3_-N was negatively associated, to a high extent, with the mesothelium thickness, and, to a medium extent, with the thickness of the muscular layer. The concentrations of individual SCFAs and total SCFAs were generally positively linked to the parameters related to the rumen papillae and rumen wall. Notably, the concentration of butyrate showed an extremely high association with the thicknesses of the mesothelium and the muscular layer, and a slightly strong association with the thickness of the submucosa inside the rumen wall (**Figure 4O**). Generally, these findings implied that the roughage quality directly affects rumen fermentation, and potentially indirectly influences rumen development via SCFAs, especially butyrate.

### 3.4 Composition and function of the rumen microbiota are affected by the roughage quality in post-weaned Hu sheep

**Figure 5:**
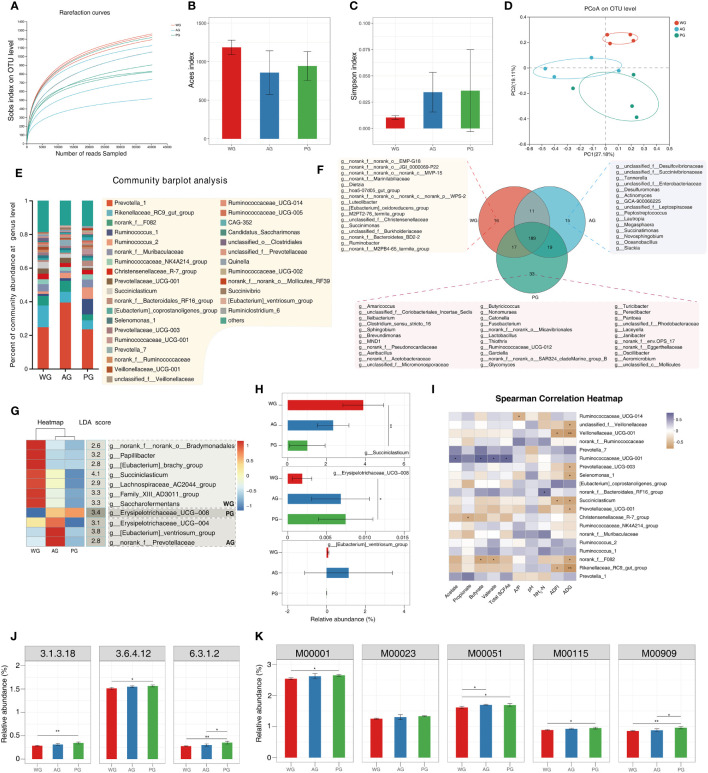
Rumen microbiota analysis and functional prediction of the microbiome from rumen fluid of post-weaned sheep. **(A)** Rarefaction curves of microbiome data measured by Sob index at the OTU level. **(B, C)** Aces index and Simpson index of microbiome data, respectively. **(D)** Principal coordinate analysis (PCoA) of the microbiome data. **(E)** Microbiome composition of ruminal fluids at the genus level (the microbes with a relative abundance ≥1%). **(F)** The shared and specific microbes in the ruminal fluids among three groups. **(G)** Signature microbes of each group identified via intersecting ANOVA and LefSE analysis. **(H)** The relative abundances of signature microbes identified for each group. **(I)** Pearson’s correlation analysis of microbe abundances with rumen fermentation parameters, ADFI, and ADG. **(J, K)** The enzymes and modules with differential abundances predicted by PICRUSt 2: 3.1.3.18, phosphoglycolate phosphatase; 3.6.4.12, DNA helicase; 6.3.1.2, glutamate–ammonia ligase; M00001, glycolysis (Embden–Meyerhof pathway), glucose → pyruvate; M00023, citrate cycle (TCA cycle, Krebs cycle); M00051, reductive citrate cycle (Arnon–Buchanan cycle); M00115, citrate cycle, second carbon oxidation, 2-oxoglutarate → oxaloacetate; and M00909, gluconeogenesis, oxaloacetate → fructose-6P.

To unearth the possible rumen microbiota-associated mechanisms underlying the differential production performances and rumen parameters among groups, we performed 16s rRNA sequencing of ruminal fluids, analyzed microbiota composition, and predicted the functional alterations associated with fluctuations in the microbiota. The rarefaction curves (indicated by the Sobs index at the OTU level) of all samples exhibited a steady pattern when the number of reads sequenced exceeded 10,000 (**Figure 5A**), thus indicating that the microbiome sequencing data were quantitatively adequate. Subsequently, we assessed the richness and diversity of microbiota in the three groups. The Aces index of the rumen fluid microbiome in the AG and PG was higher than of that in the WG, although it did not meet the statistical standard (**Figure 5B**). In contrast, the Simpson index values of the AG and PG were lower than that of the WG (**Figure 5C**). The above results hint that the roughage quality possibly alters the richness and diversity of ruminal fluid microbiota in post-weaned Hu sheep. We also performed β-diversity analysis (i.e., principal coordinate analysis, PCoA) to evaluate the general similarity of microbiota among groups. The obtained result hinted that the samples of WG are located in an individual area on the two-dimensional map, which clearly separates them from samples of the AG and PG (**Figure 5D**). The β-diversity analysis implies that the microbiota of the AG and PG share a higher similarity to that of the WG at the global level. The OTU data at the genus level are provided in **Supplementary Table 5**.

Subsequently, we characterized the microbial composition of ruminal fluids collected from post-weaned Hu sheep at the genus level. The top ranked microbes (relative abundances > 1%) include: *Prevotella 1*, *Rikenellaceae RC9 gut group, norank f F082*, *Ruminococcus 1*, *Ruminococcus 2, norank fMuribaculaceae*, *Ruminococcaceae NK4A214 group*, *Christensenellaceae R-7 group*, *Prevotellaceae UCG-001, and Succiniclasticum* (**Figure 5E**). The majority of the microbes belonged to fibrolytic bacteria (e.g., *Ruminococcus 1*), amylolytic bacteria (e.g., *Prevotella 1*), secondary metabolite utilization-related bacteria (e.g., *Succiniclasticum*), and other microbes with undefined functions (e.g., *Rikenellaceae RC9 gut group*). We also identified the specific and shared microbes that existed in the three groups. As shown in **Figure 5F**, a large proportion of the microbes (189 genera) were common to all groups. A very small number of microbes were exclusively found in the WG (e.g., *norank f norank o EMP-G18*), AG (e.g., *unclassified f Desulfovibrionaceae*), and the PG (e.g., *Amaricoccus*). Furthermore, we defined the signature microbes of each group via intersecting the results from the ANOVA and Lefse analysis (**Supplementary Table 6**). As shown in **Figure 5G**, seven microbes (including *Succiniclasticum*, *Saccharofermentans*, Family XIII AD3011 group, and *Papillibacter*) were filtered as the marker microbes for the WG. The *Eubacterium ventriosum group*, *Erysipelotrichaceae UCG-004*, and *norank f Prevotellaceae* were found as signature bacteria of the AG. *Erysipelotrichaceae UCG-008* was screened as the marker species of the PG. The relative abundances of the first-ranked microorganism for each group are displayed in **Figure 5H**. We also performed Pearson’s correlation analysis of the relative abundances of microbes with the concentrations of SCFAs and other parameters (**Figure 5I**). The results suggested that the abundances of *Ruminococcaceae UCG-014* are negatively correlated with the A/P ratio. The growth parameters (i.e., ADFI and ADG) showed negative relationships with the abundances of several microbes, including unclassified f *Veillonellaceae*, *Veillonellaceae 001*, *Prevotellaceae UCG-003*, *Selenomonas 1*, *Succiniclasticum*, *Prevotellaceae UCG-001, norank f F082*, and *Rikenellaceae RC9 gut group*. Several microbes (e.g., *Rikenellaceae RC9 gut group*) were among the top-ranked organisms of the ruminal fluid microbiota across all samples (**Figure 5E**). In addition, the level of *Ruminococcaceae UCG-001* was positively associated with the levels of all SCFAs other than propionate, suggesting a potential pivotal role of the microbe in microbial decomposition of diet carbohydrates and production of partial SCFAs. Moreover, the level of NH_3_-N exhibited a positive correlation with the norank f *Bacteroidales RF16 group*, suggesting that the microorganism participates in the microbial degradation of proteins, polypeptides, and amino acids. We also noticed that the abundance of *Christensenellaceae R-7 group* was negatively related to the content of propionate, and that the same relationship exists among *Rikenellaceae RC9 gut group* and the levels of butyrate and valerate. These analyses hinted that ruminal microbes are involved in the regulation of the rumen fermentation and animal growth, and that these relationships are influenced by forage quality.

Finally, we adopted PICRUSt2 (Douglas et al., 2020) to forecast the functional information of microbiota inside ruminal fluids. As shown in **Figures 5J, K**, several enzymes (3.1.3.18, phosphoglycolate phosphatase; 3.6.4.12, DNA helicase; and 6.3.1.2, glutamate–ammonia ligase) involved in nutrient degradation and synthesis, and five modules related to carbohydrate metabolism [e.g., M00001, glycolysis (Embden–Meyerhof pathway), glucose → pyruvate], were functionally enriched. These results implied that the forage quality changes the microbial functions related to carbohydrate metabolism. All the functional prediction results are provided in **Supplementary Table 7**.

### 3.5 Correlation and network analysis reveal interrelationship of microbes and their potential roles in rumen metabolism

**Figure 6:**
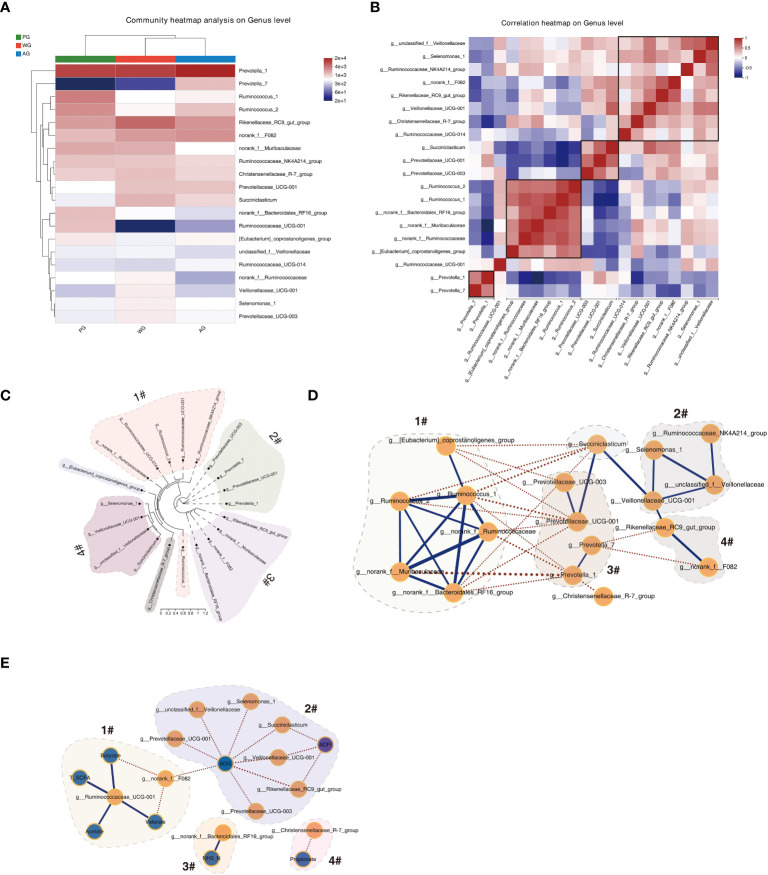
Correlation and network analysis of the microbiome and visualization. **(A)** Heat map of the top 20 microbes using relative abundances showing the clustering results of microbes. **(B)** Correlation map of the top 20 microbes showing the interrelationship among microbes. **(C)** Phylogenetic analysis of microbes using FastTree. **(D)** Visualization of the co-occurrence network among microbes. **(E)** Visualization of the correlation analysis result between the microbiome data and rumen fermentation parameters, ADG, and ADFI.

To understand the interrelationship of microbes in the rumen ecosystem and speculate their significant functions in rumen metabolism, we performed clustering, phylogenic, and correlation analyses using the OTU data of the microbes. As shown by the heat map in **Figure 6A**, the microbes with similar abundances across three groups clustered together, suggesting that these microbes possess similar functions or work synergistically to decompose polymers in the rumen. For example, *Ruminococcus 1* (containing the main cellulolytic species *Ruminococcus flavefaciens* and *Ruminococcus albus*) and *Ruminococcus 2* were gathered together, indicating that *Ruminococcus* 2 plays an important role in fiber degradation. Meanwhile, the succinate-producing bacteria (*Prevotellaceae UCG-001*) and the succinate-utilizing bacteria (*Succiniclasticum*) emerged in the same minor clade, substantiating the possible existence of a cross-feeding relationship between them. Moreover, the correlation analysis results of the bacterial abundances verified the above discoveries (**Figure 6B**). The abundances of microbes with similar functional characteristics or close metabolic interdependent relationships in the rumen were positively correlated. At the same time, the microorganisms with competitive relationships were negatively associated. For instance, the abundances of *Prevotella 1* and *Prevotella* 7 showed a positive correlation relationship, whereas the abundance of a cellulolytic bacterium (*Ruminococcus 1*) displayed a negative association with an amylolytic microbe (*Prevotellaceae UCG-001*). The above results indicated that the abundance fluctuations of ruminal bacteria were consistent with their respective roles or complex metabolic relationships in maintaining the ecosystem of rumen.

The phylogenic analysis revealed that the top 20 ranked microbes are mainly divided into four clades (**Figure 6C**). Clade 1 is mainly composed of the bacteria belonging to the Ruminococcaceae family. Clade 2 comprises the microbes belonging to the Prevotellaceae family. The microorganisms in clade 3 belong to diverse families but are in the same order (Bacteroidales). A similar observation was made for the microorganisms in clade 4, except that all the microbes are in the Selenomonadales order. The above phylogenetic study hinted that the functions of microbes in the same clade are potentially relevant.

We also visualized the relationship of microbes at a global level through cooccurrence analysis and found that a complex interaction network exists. As shown in **Figure 6D**, the abundances of the microbes in group 1 (Ruminococcaceae family) were generally negatively associated with those of the microbes in group 3 (Prevotellaceae family) and those in Succiniclasticum. In addition, the levels of four microbes in group 2 were positively linked. However, the abundances of *Rikenellaceae RC9 gut group* and *norank fF082* were negatively related to *Prevotella* 7. We also visualized the interactive map of microbes with rumen fermentation parameters and growth performance, and found that the microbes in group 2 are negatively correlated with AGD and ADFI (**Figure 6E**). The microorganisms in the groups contained amylolytic bacteria (e.g., *Prevotellaceae UCG-001*), intermediate product-utilizing bacteria (e.g., *Succiniclasticum*), and bacteria with unknown functions in the rumen (e.g., *Rikenellaceae RC9 gut group*). Moreover, the relationships of several bacterium with SCFAs and NH_3_-N were displayed. The above results demonstrated that certain microbes are potential regulators of rumen fermentation and animal growth.

### 3.6 Roughage quality impacts the DNA methylome landscape of the rumen wall

**Figure 7:**
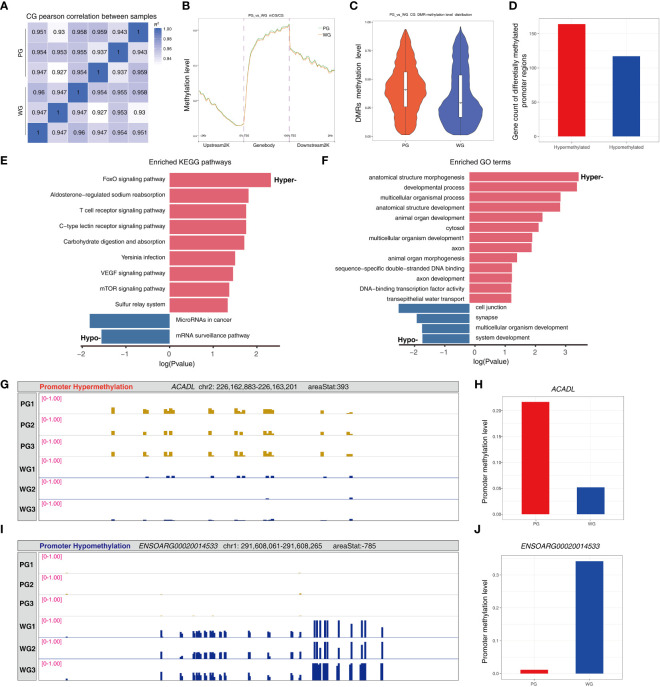
Bioinformatic analysis of rumen wall methylomes. **(A)** Pearson’s correlation analysis of methylomes among samples. **(B)** The methylation level of sites at regions of the gene body, and at the regions of upstream and downstream 2K segments of gene body. **(C)** The relative methylation level of the DMRs between groups. **(D)** The counts of genes with differentially methylated promoter regions. **(E)** KEGG pathway enrichment of genes with differentially methylated promoter regions. **(F)** GO term enrichment of genes with differentially methylated promoter regions. **(G, H)** The hypermethylation status of *ACADL* in the PG compared with the WG. **(I, J)** The hypomethylation status of *ENSOARG00020014533* in the PG compared with the WG. ACADL, acyl-CoA dehydrogenase, long chain.

Based our previous observation that the rumen development is affected by fodder quality, we performed RRBS to explore whether or not the universal epigenetic status of rumen tissues is affected by that. We obtained highly qualified data from the rumen tissues of animals in the PG and WG (**Supplementary Table 8**). Subsequently, we found that the methylation levels of the samples were highly correlated at a global level (**Figure 7A**). However, the proportions of methylated nucleotides exhibited an elevated trend in the gene body, upstream 2K, and downstream 2K regions in the PG compared with the WG (**Figure 7B**). Moreover, the methylation levels of differentially methylated regions (DMRs) in the PG were higher than in the WG (**Figure 7C**). Because the methylation status of gene promoter regions is closely related to gene expression via transcriptional regulation, we focused on the promoter segments of DMRs. A total of 164 genes with hypermethylated promoters and 117 genes with hypomethylated promoters were identified in the PG compared with the WG (**Figure 7D**; **Supplementary Table 9**). The KEGG pathway enrichment results hinted that the FoxO signaling pathway, aldosterone-regulated sodium reabsorption, T-cell receptor signaling pathway, carbohydrate digestion and absorption, VEGF signaling pathway, mTOR signaling pathway, and the sulfur relay system were overrepresented in the genes with hypermethylated promoters (**Figure 7E**). Moreover, the microRNAs in the cancer and mRNA surveillance pathways were found to be enriched genes with hypomethylated promoters. GO analysis suggested that the majority of genes with hypermethylated promoters are involved in organism development (e.g., anatomical structure morphogenesis), transcription factor functionality (e.g., DNA-binding transcription factor activity), and transepithelial water transport (**Figure 7F**; Supplementary Table). The enriched GO terms of the genes with hypomethylated promoters included cell junction, synapse, multicellular organism development, and system development. The detailed results of gene functional enrichment analysis are provided in **Supplementary Table S10**. Furthermore, we demonstrated that the promoter regions of *ACADL* and *ENSORAG0020014533* are hypermethylated and hypomethylated, respectively (**Figures 7G–J**). These results hinted that forage quality possibly determines the rumen development via altering the rumen methylation status and gene expression at an epigenetic level.

## 4 Discussion

Comprehensive investigation of the microbial and host responses to low- and high-quality forages is the theoretical foundation for developing new strategies for the efficient utilization of abundant and cheap fodders in ruminant production (Buxton et al., 1996). In this study, we assessed the growth performance differences of fattening Hu sheep fed three types of roughage and evaluated the corresponding microbiota and host variations. Our results revealed that the fodder quality determines animal production performance via affecting the microbial composition and interactions, and potentially influencing rumen development through SCFA-mediated epigenetic modification. At the same time, these observations suggested that there is an intricate fodder–microbe–host interplay in ruminant production, providing new windows to improve animal growth performance via manipulating the interactions.

The inferior production performance and meat quality of post-weaned Hu sheep fed low-quality forage-based diet are consistent with previous studies on ruminants, including cattle (Zhu et al., 2022), goats (Sun et al., 2018), and sheep (Obeidat et al., 2019). The significantly lower digestibility of DM, CP, and CF in the ration composed of low-quality forage, and concomitant longer feed retention time in the digestive tract and lower level of feed intake are the principal factors causing the undesired results (Tufail et al., 2021). These constraint factors are further determined by the inherent physical, chemical, and nutritive features of forage. For example, wheat straw, the most abundant low-quality forage, has a complex plant cell wall structure, low CP content, and a superficial wax layer, all of which prevent the physical contact of nutrient-degrading enzymes with their substrates (Tufail et al., 2021). The manual breaking of the cell wall and wax layer via hydrothermal and chemical treatments (Jackson, 1977; Yang et al., 2016), or of increasing the CP content through ammonization (Schlegel et al., 2016) have been widely adopted to enhance forage quality and animal feeding performance. Except for higher contents of conventional nutrients (e.g., CP, vitamins, and minerals), certain high-quality forage types (e.g., alfalfa hay) possess abundant natural bioactive compounds with multiple beneficial effects on animal production. In our study, we found that the animals fed an alfalfa hay-based diet exhibited better immunity and a lower inflammation index than those fed wheat straw- and peanut vine-based diets. Alfalfa saponin and flavone are the well-known master bioactive substances that are capable of boosting the immune system and antioxidation status in monogastric animals and ruminants (Chaudhary et al., 2018; Chen et al., 2020; Yang et al., 2022). The addition of alfalfa extracts or the implementation of a reasonable combination of low-quality forage with alfalfa hay are useful strategies to cut down feed cost and improve rearing incomes.

The rumen harbors a complex microbial community that can efficiently degrade the feed ingested by the host and synthesize nutrients (e.g., microbial proteins and vitamins) for the host’s own utilization. Thus, ruminal fermentation is tightly controlled by the feed composition (i.e., the ratio of concentrate to forage and the proportion of structural carbohydrate versus non-structural carbohydrates), which further impacts rumen development and host growth. We showed that the rumen fermentation and tissue morphology of Hu sheep are markedly influenced by roughage quality. Sheep fed a wheat straw-based diet possessed a higher level of NH_3_-N in their ruminal fluid than those fed high-quality forages. This result is highly consistent with the discovery made in dairy cattle (Feng et al., 1993). Lower digestibility of fermentable carbohydrates caused insufficient provision of available energy for microbial usage of NH_3_-N for protein synthesis is the underlying mechanism for that (Pathak, 2008). This viewpoint fits with the result that animals fed high-quality roughage exhibit elevated levels of SCFAs and fermented products of diet carbohydrates, compared with those fed wheat straw. In addition, we also found that the tissue morphologies of rumen papillae and wall are markedly influenced by the fodder quality. The main functions of the rumen epithelium include the absorption of fermented SCFAs as a primary energy source for host maintenance, and rumen development is also drastically stimulated by SCFAs, especially butyrate (Dieho et al., 2016; Niwińska et al., 2017). In our study, we found that the pH of ruminal fluid is negatively correlated with the rumen papillae surface area, which coincides with the rule that a larger surface area results in the faster transportation of SCFAs by rumen papillae. Moreover, the positive correlations of ruminal papillae parameters with the levels of individual and total SCFAs suggest the stronger ability of blood veins to transport SCFAs is facilitated by higher levels of of trophic, hormonal, and mitogenic agents that promote rumen development (Membrive, 2016).

The population-level alteration of the ruminal microbiome is the responsive action of the sophisticated ecosystem to the feed consumed by animals. We found that the community richness, diversity, and similarity of microbiomes from the ruminal fluid are similar in high-quality fodder-based diets compared with low-quality hay-based ration. The main microbes at the genus level include amylolytic bacteria (e.g., *Prevotella 1*), fiber-decomposing bacteria (e.g., *Ruminococcus 1* and *Ruminococcus 2*), and rumen secondary fermentation-related bacteria (e.g., *Succiniclasticum*). Such a pattern of sheep ruminal microbiota composition is similar to our previous report on cattle (Zhu et al., 2022). We also defined the signature microbes in each group by intersecting the results of the ANOVA and LDA analyses. *Succiniclasticum*, the microbe that was more abundant in the WG than in other groups, is involved in converting the fermented intermediate, succinate, to the final product propionate, a unique energy source (van GYLSWYK, 1995). *Succiniclasticum* also acts as a starch decomposer to produce acetate, propionate, and succinate, and further transforms succinate into propionate (Liang et al., 2021). Its relatively higher abundance in the WG is in accordance with the fact that starch in the WG diet is easier to degrade because of the accessibility and degradability of cellulose and hemicellulose being lower in wheat straw than in high-quality forages (Obeidat et al., 2019). In addition, we found that the abundances of several bacteria are closely related to rumen fermentation parameters and growth performance. For example, *Ruminococcaceae* UCG-001, the fibrolytic and cellulolytic bacteria in rumen (Thoetkiattikul et al., 2013), is positively associated with acetate, butyrate, valerate, and total SCFAs. Furthermore, most of the predicted functional modules are related to carbohydrate metabolism (e.g., glycolysis), further substantiating the notion that differences in fodder quality affect structural and non-structural carbohydrate metabolism by microbes in rumen.

As an evolutionarily complex biological system, the rumen harbors numerous microbes with competitive or cooperative relationships to fulfill the task of providing the host with microbe-derived nutrients. At present, the functions and roles of the main microbes with crucial roles in degrading main feed components (i.e., fiber, starch, and protein) have been validated. For instance, *Ruminococcus* and *Prevotella* are the bacteria that play roles of primary importance in decomposing feed fiber and starch in the rumen, respectively (Stevenson and Weimer, 2007; Dassa et al., 2014). The negative association of their abundances in the rumen have been observed in our previous study (Chuang et al., 2020). Linking the less-studied microbes with the microbes with well-studied functionalities is an effective strategy for determining the potential corresponding functions. *Rikenellaceae* RC9 gut group and norank f F082 are the top ranked microbes in the rumen, and their abundances are negatively associated with that of *Prevotella* 7, the saccharolytic bacteria (Tett et al., 2021). This observation hints that the two microbes are involved in the degradation of feed fiber in the rumen, which agrees with the elevated levels of *Rikenellaceae* RC9 gut group found in the gut microbiota of monogastric animals fed a high-fiber diet (Qiu et al., 2022; Gao et al., 2023). The clear roles of *norank f F082* in the rumen metabolism have not yet been reported yet, but its abundance in the rumen fluctuates with the change in concentrate-to-forage ratio and the type of silage (Han et al., 2022; Yi et al., 2022). Based on our correlation and phylogenetic analysis, we posit that *norank f F082* is a potential fiber degrader. The abundance of another top-ranked bacteria, norank f *Muribaculaceae*, is positively associated with fibrolytic bacteria (e.g., *Ruminococcus 2*), and is adversely correlated with amylolytic bacteria (*Prevotella 7*). Taking the above result and the closer phylogenetic relationship with *Rikenellaceae RC9 gut group* into consideration, it is reasonable to assume that norank f *Muribaculaceae* performs a fibrolytic function in the rumen. The above hypothesis is supported by the finding that the addition of polysaccharides enhanced its microbial abundance in mice gut (Yang et al., 2022). Moreover, we also found that several amylolytic and secondary fermented metabolites-utilizing bacteria are negatively correlated to ADFI and ADG. Our result conflicts with the findings that certain bacteria (e.g., *Prevotella* and *Succiniclasticum*) show positive responses to the increment of feed concentrate in goats and yaks (Ma et al., 2021; Dai et al., 2022), which usually result in better livestock growth performance. These phenomena could be explained by the fact that, with the concentration-to-forage ratio in our experiment being the same in both diets, the speed of amylolysis was faster than that of crude fiber degradation in animals fed a low-quality forage-based diet than in those a high-quality fodder-based diet, resulting in the relatively higher abundance of these bacteria. Thus, understanding the mutual relationships of microbes inside the rumen and the subsequent development of direct-fed microbes (whether isolated from the rumen or not) for the highly efficient degradation of feed fiber should be a promising strategy for improving the nutritive values of low-quality fodders.

As the organ with unparalleled structure and function, the rumen not only provides an anaerobic niche for the bacterial fermentation of ingested feed, but also transports the fermented products for the host’s usage. Thus, rumen development is tightly controlled by the feed composition (e.g., the concentrate-to-forage ratio and fodder quality) and the concentration of SCFAs, the main energy supplier and growth stimulator from the microbial degradation of feedstuff carbohydrates. We exhibited that fodder quality significantly affects both the concentration of SCFAs and the rumen morphology. We further showed that the DNA methylomes of rumen epithelia were distinct between the WG and the AG, suggesting that changes in the epigenetic modification might participate in rumen development. A previous study found cattle fed grains exhibited a relatively higher DNA methylation level of rumen tissues than those fed grass (Li et al., 2019). A higher percentage of concentrate in ruminants’ diet usually leads to elevated SCFA levels and more robust rumen wall development (Suárez et al., 2007), which is highly consistent with our observations that the diet containing high-quality fodders boosted rumen papillae growth. We also found that the global DNA methylation levels of rumen tissues were higher in the PG than in the WG, which agrees with the levels of ruminal SCFAs. Based on the above facts, we propose that SCFA-mediated DNA methylation modification is involved in rumen development. In addition to signaling through binding membrane receptors and regulating the activity of histone deacetylases, it has been found that SCFAs impact the methylation status of genes in multiple animal tissues or cell lines (Lu et al., 2018; Chang et al., 2019; Guo et al., 2022). We also performed the functional enrichment of genes with differentially methylated promoters and discovered that several signaling pathways pertaining to rumen development are significantly enriched. For example, it has been reported that the FoxO signaling pathway and mTOR signaling pathway are the key signaling cascades involved in rumen development (Lin et al., 2019; Pacífico et al., 2022). Moreover, the GO enrichment analysis revealed that the majority of enriched terms are related to tissue or organ development. These results further demonstrate that DNA methylation-mediated epigenetic modification is a crucial factor for rumen development, as previously reported in other tissues (Jin and Liu, 2018). We also screened two genes with promoter regions that were hypermethylated or hypomethylated in the PG compared with the WG. It has been reported that *ACADL*, the gene with elevated promoter methylation level in the PG, is a tumor suppressor (Zhao et al., 2020). The hypomethylated gene *ENSOARG00020014533*, which encodes carbonyl reductase [NADPH] 1, regulates a wide array of biological processes (e.g., carcinogenesis) via catalyzing the reduction of carbonyl compounds, including quinones (Yokoyama et al., 2023). Although the exact roles of both the genes in rumen epithelium development have not been examined, the varied promoter methylation levels hint that they participate in rumen development. Subsequent validations of the above hypothesis and experimental examination of whether their promoter methylation is regulated by SCFAs will provide novel insights into how rumen development is directly regulated by the epigenetic modification of the host, and indirectly regulated by microbe-sourced SCFAs. Understanding the interaction relationship among ingested feed, rumen microbes, and host development will further facilitate the design of tailored strategies to precisely control rumen development, as the administration of sodium butyrate to stimulate rumen development on cattle (Górka et al., 2011).

## 5 Conclusion

Forage quality determines sheep production performance via directly influencing rumen fermentation and microbiome composition, and indirectly affecting rumen development. In terms of immune-boosting and anti-inflammation properties, alfalfa hay has marked advantages over wheat straw and peanut vine. Extensive microbial interactions (cooperative or competitive) exist among rumen microbes, and the relative abundances of certain microbial species are closely related to animal growth performance and rumen fermentation parameters. In addition, DNA methylation-mediated epigenetic modification mighty participate in the regulation of rumen development. Ongoing investigations of how the ruminal microbes and rumen respond to low-quality fodders will shine new light on strategies for lowering the feeding cost of ruminants and improving animal production.

## Data availability statement

The data presented in the study are deposited in the NCBI repository, accession numbers PRJNA991257 and PRJNA991642.

## Ethics statement

The animal studies were approved by Animal Welfare and Ethics Committee of Henan Agricultural University. The studies were conducted in accordance with the local legislation and institutional requirements. Written informed consent was not obtained from the owners for the participation of their animals in this study because we have oral agreement of consent, and we are good friends for animal rearing.

## Author contributions

SM: Writing – original draft, Writing – review & editing, Conceptualization, Formal analysis. ZL: Conceptualization, Data curation, Writing – review & editing. YZ: Conceptualization, Methodology, Project administration, Writing – original draft, Writing – review & editing. BL: Resources, Writing – review & editing. ZW: Methodology, Resources, Writing – review & editing. YC: Conceptualization, Formal Analysis, Resources, Writing – review & editing. CW: Funding acquisition, Supervision, Writing – review & editing. DL: Formal analysis, Project administration, Resources, Writing – review & editing. YS: Funding acquisition, Supervision, Writing – review & editing. MG: Investigation, Writing – review & editing.
