# Supplementary material for: Roughage quality determines the production performance of post-weaned Hu sheep via altering ruminal fermentation, morphology, microbiota, and the global methylome landscape of the rumen wall
Source: Front Microbiomes. 2024 Jan 3;2:1272625. doi: 10.3389/frmbi.2023.1272625 (PMC12993644; doi:10.3389/frmbi.2023.1272625)
Supplement: Supplementary file 1 [file DataSheet_1.zip › Supplementary Tables 1-4.DOCX]

Table S1 Internal organ index of post-weaned Hu sheep

| Items | Groups | | |
| --- | --- | --- | --- |
|  | WG | AG | PG |
| Heart（%） | 0.37±0.03 | 0.38±0.01 | 0.42±0.05 |
| Liver（%） | 1.79±0.25 | 1.77±0.17 | 1.97±0.08 |
| Spleen（%） | 0.14±0.01 | 0.15±0.02 | 0.13±0.02 |
| Kidney（%） | 0.34±0.03 | 0.35±0.03 | 0.37±0.05 |
| Lung（%） | 1.42±0.24 | 1.31±0.20 | 1.29±0.17 |

Table S2 Effects of roughage quality on slaughtering performance of post-weaned Hu sheep

| Items | WG | AG | PG |
| --- | --- | --- | --- |
| Live weight before slaughter, kg | 39.33±1.08^b^ | 39.73±2.66^b^ | 43.1±1.32^a^ |
| Dressing Percentage, % | 47.12±1.05^b^ | 49.54±2.31^ab^ | 49.99 ±2.7^a^ |
| Net meat percentage, % | 33.45±0.31^b^ | 38.00±0.43^a^ | 38.29±0.26^a^ |
| Bone/Meat | 0.32±0.25^a^ | 0.27±0.35^a^ | 0.25±0.01^b^ |
| GR value, mm | 2.45±2.45 | 2.20±0.63 | 2.23±0.80 |
| Lion eye muscle area, cm² | 24.24±4.87 | 25.83±6.71 | 25.86±5.83 |

Table S3 Effects of roughage quality on meat characteristics of post-weaned Hu sheep

| Items | WG | AG | PG |
| --- | --- | --- | --- |
| pH_45min_ | 6.60±0.29 | 6.43±0.33 | 6.52±0.25 |
| Drip loss, % | 2.53±0.58a | 1.58±0.33b | 1.37±0.11b |
| Cooked meat yield, % | 50.56±1.45 | 50.68±2.59 | 49.69±0.57 |
| Shearing force, N | 81.25±1.94a | 63.77±1.72b | 73.06±7.45a |
| Water holding capacity, % | 13.94±1.91 | 15.00±1.23 | 14.97±2.75 |
| *L** | 42.17±1.58b | 41.11±1.15b | 45.61±0.40a |
| *a** | 21.35±0.74ab | 20.47±1.16b | 21.48±0.18a |
| *b** | 6.75±0.24b | 4.39±0.31c | 7.22±0.22a |

Table S4 Rumen fermentation parameters of post-weaned Hu Sheep fed three types of diet

| Items | WG | AG | PG |
| --- | --- | --- | --- |
| pH | 6.69±0.09 | 6.67±0.08 | 6.53±0.19 |
| NH_3_-N, mg/dL | 25.19±6.20^a^ | 13.05±5.34b | 17.90±6.83ab |
| Acetate, ug/g | 35.02±0.77b | 33.92±2.70b | 45.06±2.22a |
| Propionate, ug/g | 15.21±0.47b | 17.28±1.26a | 17.97±0.42a |
| Butyrate, ug/g | 6.04±0.74b | 5.37±0.33b | 11.21±0.46a |
| Valerate, ug/g | 23.70±2.68b | 26.00±1.67b | 38.96±1.27a |
| Total VFA, ug/g | 79.91±4.27b | 88.44±6.57b | 112.12±6.53a |
| A:P | 2.31±0.14^a^ | 1.96±0.05^b^ | 2.51±0.11^a^ |
